# Safety and Efficacy of Eslicarbazepine Acetate in Children Diagnosed With a Focal Seizure Disorder: A Systematic Review and Meta‐Analysis

**DOI:** 10.1002/brb3.71036

**Published:** 2025-11-24

**Authors:** Neha Bhagwan Das, Muhammad Zain Ul Haq, Eiman Anwar, Manav Das, Ariba Asif, Umaimah Naeem, Mohammad Mohsin Khan, Mahrukh Arshad, Saima Javed, Aymar Akilimali

**Affiliations:** ^1^ Department of Medicine Peoples University of Medical and Health Sciences for Women Nawabshah Pakistan; ^2^ Department of Medicine Dow University of Health Sciences Karachi Pakistan; ^3^ Department of Medicine Liaquat University of Medical and Health Sciences Jamshoro Pakistan; ^4^ Department of Medicine King Edward Medical University Lahore Pakistan; ^5^ Department of Medicine Jinnah Sindh Medical University Karachi Pakistan; ^6^ Department of Medicine Fatima Jinnah Medical University Lahore Pakistan; ^7^ Department of Research Medical Research Circle Goma DR Congo

**Keywords:** adverse effects, eslicarbazepine acetate, focal seizures, pediatric epilepsy, treatment outcome

## Abstract

**Background:**

Epilepsy can have a negative impact on cognitive and social functioning. Eslicarbazepine acetate (ESL) is used to treat focal seizures, although its safety and efficacy, particularly as an additional treatment, warrant more study. The purpose of this study was to evaluate the safety and efficacy of ESL in children with focal seizures by analyzing data from relevant clinical trials.

**Methods:**

This study was conducted in line with the PRISMA guidelines and has been registered on the PROSPERO 2024 CRD42024569119 register. A detailed search up to July 2024 through Cochrane CENTRAL, PubMed/MEDLINE, and Google Scholar was conducted for the studies comparing ESL with placebo. For the meta‐analysis, the Mantel–Haenszel random‐effects model was applied, and heterogeneity was measured using the *I* ^2^ index.

**Results:**

The study included a total of 742 children from the randomized controlled trials; of these, 414 were given ESL, whereas 328 were given a placebo. ESL significantly reduced seizure frequency when compared to placebo (SMD = −0.29; 95% CI [−0.57, −0.02]). Nevertheless, it has been related to a greater risk of side effects, including headaches, sleepiness, nausea, vomiting, double vision, and dizziness. Adverse events were measured to evaluate clinical significance. Treatment‐emergent‐related adverse events (TEAEs) occurred in 52.2% of ESL‐treated participants versus 49.1% in placebo individuals (RR = 1.08; 95% CI [0.96–1.20]; *p* = 0.20), indicating no statistically significant increase overall. However, specific adverse events were significantly more common with ESL, including somnolence: RR = 1.95; 95% CI (1.11–3.43); *p* = 0.02 and diplopia: RR = 4.34; 95% CI (1.60–11.78); *p* = 0.004. Other common but nonsignificant adverse events included headache (11.49%), vomiting (6.15%), nausea (3.34%), and dizziness (3.2%). Adverse event‐related treatment discontinuation was reported in 5.2%–5.9% of ESL patients compared to 2.3%–2.5% of placebo patients. Most adverse events were mild to severe, although serious adverse events (e.g., status epilepticus, device‐related problems) were more common in the ESL group.

**Conclusion:**

Though ESL increases the risk of adverse effects, it dramatically reduces seizure frequency in children with focal epilepsy. These risks require regular monitoring. Subsequent study should focus on long‐term effectiveness, safety, and ways to mitigate the negative effects of ESL.

## Graphical abstract

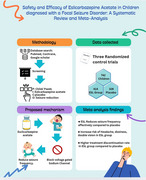

## 1 Introduction

Epilepsy comprises a range of neurological conditions marked by a consistent inclination to undergo epileptic seizures (Perucca et al. 2020). Children with epilepsy experience varying degrees of attention problems when orienting toward external stimuli and deficits in internalization hampering their cognitive processing, leading to unfavorable risk outcomes not only at psychosocial levels but also extending suitably into adulthood. In addition, these conditions are often accompanied by cognitive impairments (Jóźwiak et al. 2018). In developed nations, epilepsy affects approximately 3%–4% of the population, with up to 10% of individuals having a seizure during their lifetime. Conversely, in low‐ and middle‐income countries, the lifetime prevalence of epilepsy is significantly higher. Around 80% of people with epilepsy reside in these regions (Hesdorffer et al. 2011; Espinosa‐Jovel et al. 2018). According to Sankar et al. (2020), epilepsy often starts in early childhood, typically between 5 and 9 years of age, and children and adolescents bear substantial burdens in terms of years lived with disability and years of life lost due to disability. Refractory seizures can increase the chance of negative long‐term academic, psychological, and social effects (Dwivedi et al. 2017) and may make epilepsy patients more likely to die suddenly and unexpectedly (Donner et al. 2017). Therefore, it is essential to assess the safety of anti‐seizure medications (ASMs) comprehensively, considering their potential impacts on cognitive development, behavioral outcomes, and overall quality of life in children with epilepsy.

Despite the wide array of ASMs available, around 30% of patients experience either drug resistance or encounter significant adverse effects (Verrotti et al. 2014). Current guidelines recommend selecting ASM based on the type of seizures or syndrome, patient age, concurrent medications, and the tolerability, safety, and efficacy to optimize treatment outcomes and minimize adverse effects for individuals with epilepsy (Ferreira and Mestre 2009). Eslicarbazepine acetate (ESL) is an ASM belonging to the dibenzoazepine family. It is a single‐enantiomer compound and functions as a blocker of voltage‐gated sodium channels (VGSCs) (Benes et al. 1999). ESL is approved in the European Union for treating focal seizures (formerly known as partial‐onset seizures) with or without secondary generalization. It can be used as monotherapy in adults newly diagnosed with epilepsy and as adjunctive therapy in adults, adolescents (> 12 years old), and children (> 6 years old). In the United States, ESL is approved for both monotherapy and adjunctive therapy for treating focal seizures in patients aged 4 years and older (Veggiotti et al. 2022). However, this analysis only included randomized controlled trials (RCTs) in which ESL was evaluated as an adjuvant therapy in pediatric patients. No monotherapy trials met the investigation's inclusion criteria. As a result, the safety and efficacy findings provided here specifically address the use of ESL as an add‐on treatment to current antiepileptic prescription regimens in children and adolescents with focal epilepsy. Zhu et al. have reviewed the various adverse events associated with the use of ESL in either high doses or as an adjunctive therapy for epilepsy. The adverse events include blurred vision, diplopia, vertigo, nausea, vomiting, fatigue, dizziness, somnolence, headache, rash, dysarthria, hyponatremia, and increased gamma‐glutamyltransferase (Zhu et al. 2020). Epilepsy is especially challenging for children. When seizures begin between the ages of 5 and 9—a key time for brain development—they can interfere with learning, attention, behavior, and emotional growth. This can lead to unexpected side effects or reduced effectiveness. Because there are relatively few medications with proven safety for children, doctors must be cautious when prescribing. Treating epilepsy in children is different from treating it in adults in many ways. The developing brain is extremely susceptible to seizures and ASMs, with specific treatments, such as valproate, associated with enduring cognitive and behavioral adverse effects (Meador et al. 2013). Numerous children with epilepsy furthermore experience developmental delays, intellectual disabilities, and behavioral or psychiatric comorbidities (Ackermann and Wilmshurst 2015). These disorders impair seizure treatment and quality of life, requiring comprehensive care that incorporates medical, psychological, educational, and familial support techniques. Also, there are not many clinical trials for children, so doctors have to guess based on adult data, which makes it harder to choose the right therapy and dose. About 30% of kids with epilepsy do not respond to drugs, so they often need other treatments like ketogenic diets or surgery, which come with their own hazards and need collaboration between other fields. This inquiry was conducted to answer the particular research question: “Does ESL, when used as adjunctive therapy, reduce seizure frequency and improve response rates in children with focal seizures compared to placebo?” We hypothesized that ESL, when used as an adjunctive therapy, would decrease the standardized seizure frequency (SSF) and increase the proportion of patients achieving ≥ 50% seizure reduction, without a substantial increase in serious adverse events (SAEs) compared to placebo.

## 2 Methods

This study has been registered on PROSPERO which can be cited as https://www.crd.york.ac.uk/prospero/display_record.php?ID=CRD42024569119.

### 2.1 Research Retrieval Strategy

The study results were presented in accordance with the recommendations of the Preferred Reporting Items for Systematic reviews and Meta‐Analysis (PRISMA) statement (Moher et al. 2009).

We conducted a thorough electronic database search for studies on the safety and efficacy of ESL in children with focal seizure disorder, utilizing MEDLINE (via PubMed), Cochrane Central Register of Controlled Trials (CENTRAL), and Google Scholar. The Medical Subject Headings (MeSH) and keywords used included: (“Child”[MeSH] OR “Child, Preschool”[MeSH] OR “Paediatrics”[MeSH] OR “Infant”[MeSH] OR “Child, Hospitalized”[MeSH] OR “Children”[MeSH]) AND (“Eslicarbazepine Acetate”[MeSH] OR “Eslicarbazepine Acetate”[Title/Abstract] OR “ESL”[Title/Abstract]) AND (“Epilepsies, Partial”[MeSH] OR “Seizures”[MeSH]).

Detailed search strategy is given in Table S1.

### 2.2 Inclusion and Exclusion Criteria

Studies were eligible for inclusion if they met the following criteria: RCT design; participants aged 2–18 years with a diagnosis of focal seizure disorder; ESL as an adjunctive therapy; comparison to placebo or an active comparator; and assessment of seizure frequency, responder rate, or adverse events.

Studies were excluded if they employed a design other than RCT; included participants outside the specified age range; focused on animal or in vitro research; solely examined pharmacokinetics or drug interactions; used ESL as a monotherapy lacked sufficient data for analysis; were published in languages other than English; or represented reviews, case reports, or observational studies.

### 2.3 Outcomes

The primary outcomes of this study were relative reduction in SSF from baseline and the proportion of patients achieving a ≥ 50% reduction in standard seizure frequency (responders). SSF was defined as the number of seizures per 28 days, adjusted from the total seizure count during the treatment period. The secondary outcome was the incidence of treatment‐emergent adverse events (TEAEs). SSF was calculated as the percentage change in seizure frequency from baseline to the end of the study period. TEAEs were defined as any adverse event occurring after treatment initiation and considered possibly, probably, or related to the drug.

### 2.4 Data Extraction

To ensure data accuracy and minimize bias, standardized procedures guided the data extraction process. A pilot‐tested data extraction form was developed to collect relevant information from included studies. Two independent reviewers (Z.H. and N.B.) screened titles and abstracts, followed by full‐text assessments of potentially eligible studies. Disagreements at any stage were resolved through discussion or involving a third reviewer (A.A.). The data extraction form captured the following key information:

- Study characteristics (author, publication year, country, study design, sample size, participant demographics)
- Intervention and comparator details (dosage, duration, etc.)
- Outcome data (seizure frequency, responder rate, adverse events)
- Other relevant data (duration of follow‐up, concomitant medications)

### 2.5 Bias Assessment and Outcome Validity

The methodological quality of included RCTs was assessed using the Cochrane Risk of Bias 2 (Rob 2) tool. This tool evaluates five domains: randomization, deviations from intended interventions, missing outcome data, measurement, and reporting bias. Two independent reviewers conducted the risk of bias assessment. Discrepancies were resolved through consensus or by a third reviewer.

The identified studies were assessed for bias using the Cochrane Collaboration's criteria (Higgins and Green 2011).

Traffic light plot and weighted bar plot are provided in Figures S1 and S2, respectively.

### 2.6 Statistical Analysis

A random‐effects model was employed to synthesize data and estimate pooled effect sizes for primary and secondary outcomes. Effect sizes were expressed as risk ratios (RR) or mean differences with 95% confidence intervals (CI). Heterogeneity was assessed using the *I* ^2^ statistic, with values categorized as negligible (0%–40%), moderate (30%–60%), substantial (50%–90%), or considerable (75%–100%). Funnel plots and appropriate statistical tests, used to assess publication bias, were not used due to the scarcity of included RCTs. To ensure the focus on the target population, data analysis was restricted to children aged 2–18 years. Dose–response relationships and long‐term outcomes were explored when feasible. The quality of evidence was assessed using the GRADE approach. All statistical analyses were performed using Review Manager (RevMan) software.

## 3 Results

### 3.1 Search Results

Nine hundred thirty‐seven records were identified by searches of the databases and trial registers. Five RCTs were retrieved for detailed assessment. Four of the five RCTs identified were not included in the analysis. The primary reason for exclusion was that the studies did not meet the predefined Population, Intervention, Comparison, and Outcome (PICO) criteria, making them ineligible for inclusion in the review. Furthermore, two RCTs were included in the previous version of the review. In this analysis, one more RCT is included. Thus, three studies were considered in the review, and all three were included in this meta‐analysis (Jóźwiak et al. 2018; Kirkham et al. 2020; Mintz et al. 2020) (Figure S3).

### 3.2 Characteristics and Risk of Bias of Included Studies

All three studies were randomized, double‐blind, placebo‐controlled, multicenter, parallel‐group trials as shown in Table S2. In a study by Kirkham et al. (2020), the recommended “target” dose of the 12‐week maintenance period was 20 mg/kg/day. In a study by Jóźwiak (2018), patients received ESL 30 mg/kg/day. The studies included *three RCTs comprising* 742 participants, 414 allocated to ESL and 328 to placebo. Majority of the percentage of the participants belonged to Caucasian ethnicity. All the trials employed centralized randomization procedures along with appropriate strategies for sequence generation and allocation concealment. Overall bias in all three RCTs was found to be at low risk. Randomization process shows low risk of bias after assessment across all domains using the Cochrane Rob 2 tool. Since none of the studies were rated as high, sensitivity analysis excluding high‐risk studies was deemed unnecessary.

Missing outcome data, deviations from reported interventions, and selection of the reported result all show low risk of bias after assessment. Studies characteristics are shown in Table S3.

Baseline characteristics and outcomes of the participants are provided in Tables S4 and S5, respectively.

### 3.3 Relative Change in Standardized Seizure Frequency and Response Rate at Any Dose

**FIGURE 1:**
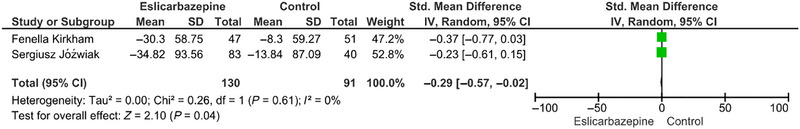
Relative change in standardized seizure frequency and response rate at any dose.

The Standardised Seizure Frequency (SSF) between the active and placebo arms varied across the trials during the treatment maintanance period. In particular, ESL users experienced fewer seizures than placebo users (df = 1, p = 0.04, Chi squared = 0.26; I2 = 0%), as indicated by the standardised mean difference (SMD) of ‐0.29 95% CI [‐0.57, ‐0.02] *p* = 0.04, Chi‐squared = 0.26; *I* ^2^ = 0%) (Figure 1). The combined analysis showed that people who took ESL had fewer measured seizures during the maintenance period of treatment compared to people who took a placebo (SMD = −0.29; 95% CI [−0.57 to −0.02]). The data for heterogeneity showed that there was not a lot of disagreement between the studies (Chi‐squared = 0.26, df = 1, *p* = 0.61; *I* ^2^ = 0%). If the *I* ^2^ value is 0%, it means that there was no observed heterogeneity. This means that the treatment result was the same in all of the studies that were looked at. This makes the pooled estimate more reliable and backs up the finding that ESL has a small but statistically significant effect on lowering the number of seizures.

### 3.4 Treatment‐Related Adverse Event

**FIGURE 2:**
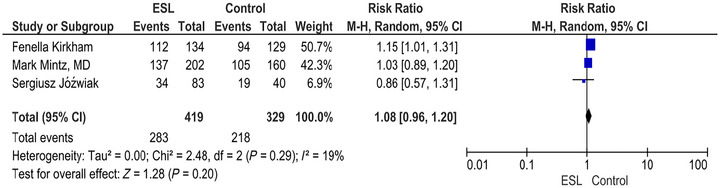
Treatment‐related adverse events.

The overall RR to develop at least one treatment‐related adverse event, not including SAEs, during treatment was 1.08 (95% CI [0.96, 1.20]; *p* = 0.20). *I* ^2^ = 19% as shown in the forest plot in Figure 2.

The percentage incidence of various adverse effects includes headache 11.49% in the ESL group versus 9.12% in the placebo group (RR 1.26; 95% CI 0.70–2.26; *p* = 0.44). Similarly, somnolence occurred in 7.35% of ESL‐treated patients compared to 3.75% in placebo (RR 1.95; 95% CI 1.11–3.43; *p* = 0.02), while other adverse events depicted the percentage incidence of vomiting 6.15%, nausea 3.34%, diplopia 3.74%, and dizziness 3.2% as shown in Table S6.

### 3.5 Headache

**FIGURE 3:**
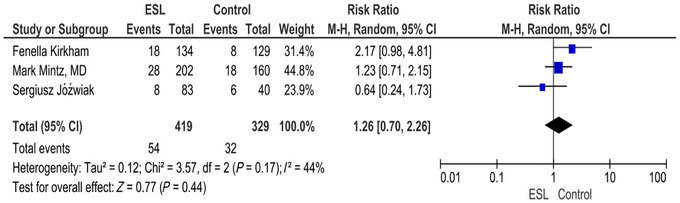
Headache.

According to the forest plot, RR was found to be 1.26 (0.70, 2.26) (*p* = 0.44) with a CI of 95% and *I* ^2^ of 44%. There was no significant difference in the number of events with headache in the intervention and placebo arms. The intervention does not appear to have a clear effect on headache incidence based on the available data (Figure 3).

### 3.6 Somnolence

**FIGURE 4:**
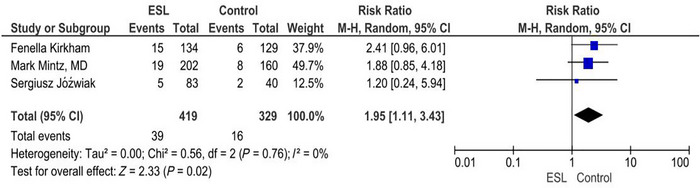
Somnolence.

For somnolence, the forest plot reveals that the RR was found to be 1.95 (1.11, 3.43) (*p* = 0.02) and *I* ^2^ = 0%. The intervention is associated with a significantly higher risk of somnolence compared to the placebo. There is no significant heterogeneity among the studies, and the effect is considered statistically significant based on the *p* value (Figure 4).

### 3.7 Vomiting

**FIGURE 5:**
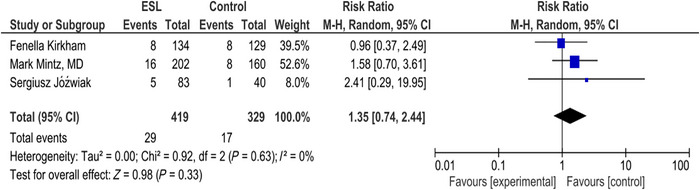
Vomiting.

In the forest plot for vomiting, RR was 1.35 (0.74, 2.44) (*p* = 0.33). There was no significant difference in the number of vomiting events in the placebo and the intervention arms (*I* ^2^ = 0%). There is no significant difference in the incidence of vomiting between the intervention and placebo groups (Figure 5).

### 3.8 Nausea

**FIGURE 6:**
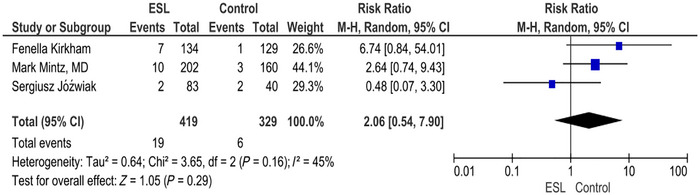
Nausea.

The forest plot for nausea shows RR of 2.06 (0.54, 7.90) (*p* = 0.29; *I* ^2^ = 45%). The intervention does not show a statistically significant effect on the risk of nausea compared to the placebo. Although the RR suggests a potential increase in nausea with the intervention, the result is not statistically significant (Figure 6).

### 3.9 Diplopia

**FIGURE 7:**
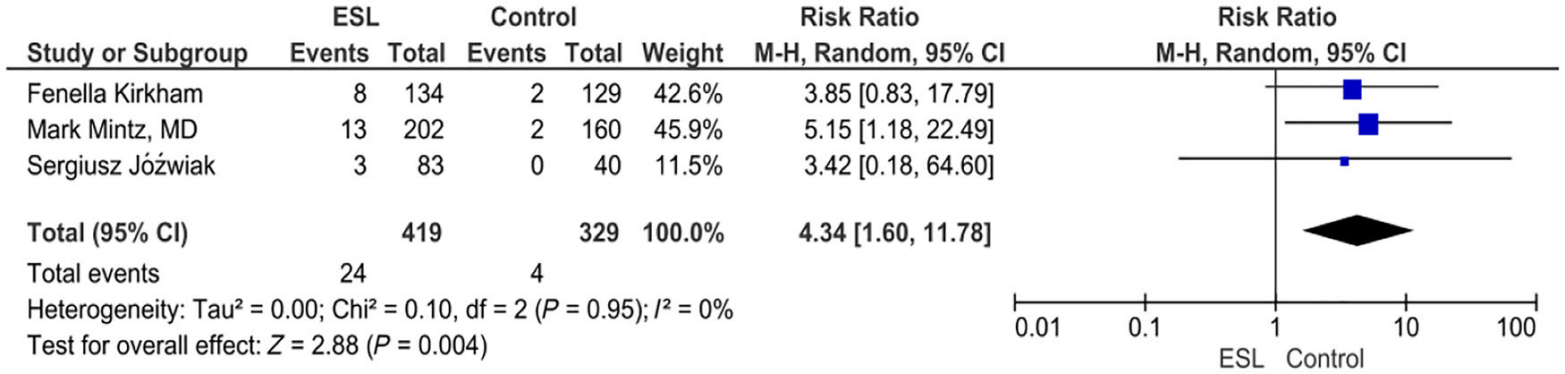
Diplopia.

The forest plot for diplopia shows RR of 4.34 (1.60, 11.78) (*p* = 0.004; *I* ^2^ = 0%). The intervention is associated with a significantly increased risk of diplopia compared to the placebo. The result is statistically significant, and there is no heterogeneity among the studies (Figure 7).

### 3.10 Dizziness

**FIGURE 8:**
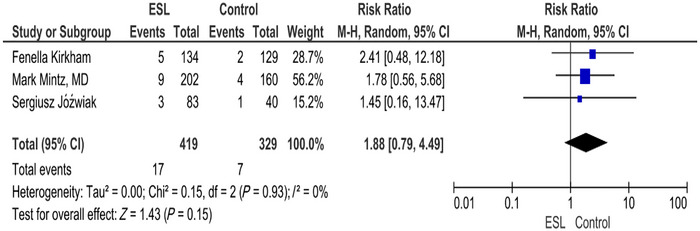
Dizziness.

The forest plot for dizziness shows RR of 1.88 (0.79, 4.49) (*p* = 0.15; *I* ^2^ = 0%). The intervention does not show a statistically significant effect on the risk of dizziness compared to the placebo. The result is not significant, and there is no heterogeneity among the studies (Figure 8).

### 3.11 Treatment Withdrawal

In examination by Kirkham et al., treatment was discontinued because of a TEAE in seven patients (5.2%) treated with ESL and in three patients (2.3%) in the placebo. While in inquiry by Jóźwiak et al., only five patients prematurely discontinued the drug due to the adverse effects. However, in an investigation by Mintz et al., 2.5% patients from the placebo and 5.9% of patients developed SAEs that led to the premature discontinuation of the drug.

According to the GRADE approach, all results are of high evidence quality.

## 4 Discussion

This study revealed the efficacy, safety, and tolerability of ESL when used as an adjuvant treatment for pediatric patients who present with focal seizures, with or without secondary generalization, despite concomitant therapy with one or more ASMs. All three included studies are randomized, double‐blind, placebo‐controlled trials. The participants in the studies were randomly assigned to either the ESL or placebo group. These studies were multicenter and involved parallel groups, as detailed in Table 1. A survey by Fenella Kirkham had a targeted dose of 20 mg/kg/day for a 12‐week maintenance period. However, a study by Sergiusz Jóźwiak administered ESL at a higher dose (30 mg/kg/day) to the patients. Collectively, these studies involve 742 participants, 414 of whom received ESL and the remaining 328 a placebo. The participants predominantly consisted of individuals of Caucasian descent. All three studies used centralized randomization to prevent selection bias. Allocation concealment was also used to reduce the risk of bias. Overall bias assessment in these RCTs indicates a low risk, suggesting that the results are appropriate; however, a sensitivity analysis excluding high‐risk studies was not performed. The randomization process was assessed and also found to be at low risk of bias. Furthermore, the trials demonstrated a low risk of bias regarding missing outcome data, deviations from the planned interventions, and the selection of reported results, indicating that the trials were conducted according to protocol and that the reported findings are likely to be accurate reflections of the effects being measured.

**TABLE 1:** Research retrieval strategy.

**Databases**	**Search strategy**	**Filters applied**	**Results**
PubMed	("Child"[MeSH Terms] OR "child, preschool"[MeSH Terms] OR "Pediatrics"[MeSH Terms] OR "Infant"[MeSH Terms] OR "child, hospitalized"[MeSH Terms]) AND ("eslicarbazepine acetate"[Title/Abstract] OR "ESL"[Title/Abstract] OR "eslicarbazepine acetate"[Title/Abstract]) AND ("epilepsies, partial"[MeSH Terms] OR "Seizures"[MeSH Terms] OR "epilepsy, partial, sensory"[MeSH Terms])	Randomized control trial, articles in English language and till year 2024	05
Cochrane	**#1**: MeSH descriptor: [Child] explode all trees **#2**: eslicarbazepine acetate **#3**: MeSH descriptor: [Epilepsy] explode all trees **#4**: MeSH descriptor: [Seizures] explode all trees **#5**: MeSH descriptor: [Placebos] explode all trees **#6**: MeSH descriptor: [Treatment Outcome] explode all trees **#7**: #1 AND #2 AND #3 OR #4 AND #5 AND #6	Randomized control trial, articles in English language and till year 2024	82,499 265 3515 1782 27,215 203,814 11
Google Scholar	“children” OR “pediatrics” AND “epilepsy” OR “seizures” AND “eslicarbazepine acetate” AND “placebo” AND “efficacy” OR “treatment outcome”	Randomized control trial, articles in English language and till year 2024	921

Our study found that patients receiving ESL as an adjunctive treatment experienced a statistically significant reduction in seizure frequency compared to placebo (SMD = –0.29; 95% CI: –0.57 to –0.02), indicating a modest therapeutic benefit. The fact that there was no heterogeneity (*I* ^2^ = 0%) among the studies that looked at seizure frequency shows that ESL worked in all of the trials, which supports the idea that the treatment effect seen can be used in other situations. While this confirms ESL's efficacy, its effect size appears smaller than that reported for some other adjunctive ASMs in pediatric populations. When compared to other pediatric ASMs, ESL appears to be equally effective. A meta‐analysis conducted by Cao et al. (2019) evaluating levetiracetam (LEV) as adjunctive therapy for pediatric patients with focal seizures demonstrated a significant mean reduction in weekly seizure frequency (26.8% compared to placebo; *p* = 0.0002) and a notably increased responder rate of ≥ 50% (44.6% for LEV vs. 19.6% for placebo; odds ratio 3.11, 95% CI: 1.22–8.26). A comprehensive meta‐analysis conducted by Fei et al. (2022) found that, unlike in adults, adjunctive ESL did not produce significant improvements in response or effective rates among pediatric patients with focal epilepsy (odds ratio for response: 1.76, *p* = 0.22; for effective rate: 2.17, *p* = 0.13) (Proesmans 2016). So, ESL is a good supplemental treatment, especially for youngsters who do not respond to other treatments. However, it might not function as effectively as medications like LEV. These comparisons make it clear that ASM choices need to be tailored to each patient's needs based on how well they work and how well the patient can handle them. The long‐term efficacy and safety of ESL have also been demonstrated in the pediatric population in a previous publication (Veggiotti et al. 2022). Our findings are in line with a study that found ESL to reduce the SSF compared to placebo in adults (Gil‐Nagel et al. 2009).

Adverse events reported by participants during the trials were also evaluated. The commonly experienced adverse effects included headache, somnolence, vomiting, nausea, diplopia, and dizziness, which have also been reported previously (Heo 2020). The study found that ESL increased the risk of diplopia and somnolence, but did not substantially vary from placebo in terms of headache, vomiting, dizziness, or nausea. A publication by Kirkham (Perucca et al. 2020) indicates a slightly higher risk in the ESL group: RR 1.15 (95% CI: 1.01–1.31); however, Mark Mintz, MD, reports little to no difference between the ESL and placebo group: RR 1.03 (95% CI: 0.89–1.20). Jóźwiak suggests a lower risk in the ESL group in his research; however, the wide CI indicates uncertainty, with an RR of 0.86 (95% CI: 0.57–1.31). The overall risk of experiencing at least one adverse event was slightly higher in the ESL group compared to the placebo group; however, this difference was not statistically significant (RR, 1.08). The *Z*‐score is 1.28 with a *p* value of 0.20, indicating that the overall effect is not statistically significant.

Headache was the most frequent adverse effect, affecting over 11.49% of participants. Both groups experienced headaches at similar rates, with no significant difference between the treatment and placebo groups. The overall effect is not statistically significant (*Z*‐score 0.77 with a *p* value = 0.44). The overall RR 1.26 (95% CI: 0.70–2.26) indicates a slightly higher risk in the ESL group. *I* ^2^ value of 44% suggests a moderate heterogeneity between the studies. About 7.35% of the participants reported somnolence. Patients on the treatment were more likely to experience drowsiness (RR 1.95), indicating that the treatment can cause significant sleepiness. The overall RR 1.95 (95% CI: 1.11–3.43) indicates a higher risk in the ESL group, and the CI indicates statistical significance. The RR of studies by Kirkham and Mintz suggests a higher risk of somnolence in the ESL group, whereas the study by Jóźwiak shows little or no difference.

About 6.15% of patients experienced vomiting as an adverse event. No significant difference is recorded for vomiting between the two groups. The *Z*‐score is 0.98 with a *p* value of 0.33, suggesting that the overall effect is not statistically significant.

Nausea was also reported as an adverse event by 3.34%. The overall RR suggests a higher risk of the occurrence of nausea in the ESL group. Diplopia was reported by 3.74% of participants. The overall RR of 4.34 (1.60, 11.78) indicates an increased risk in the ESL group. Hence, the interventional group can cause noticeable vision problems.

Dizziness was the least common, affecting just over 3.2% of participants. The occurrence of dizziness was similar between the treatment and placebo groups. However, a previous investigation reported dizziness as the most common TEAE (Heo 2020; Elger et al. 2017).

**FIGURE 9:**
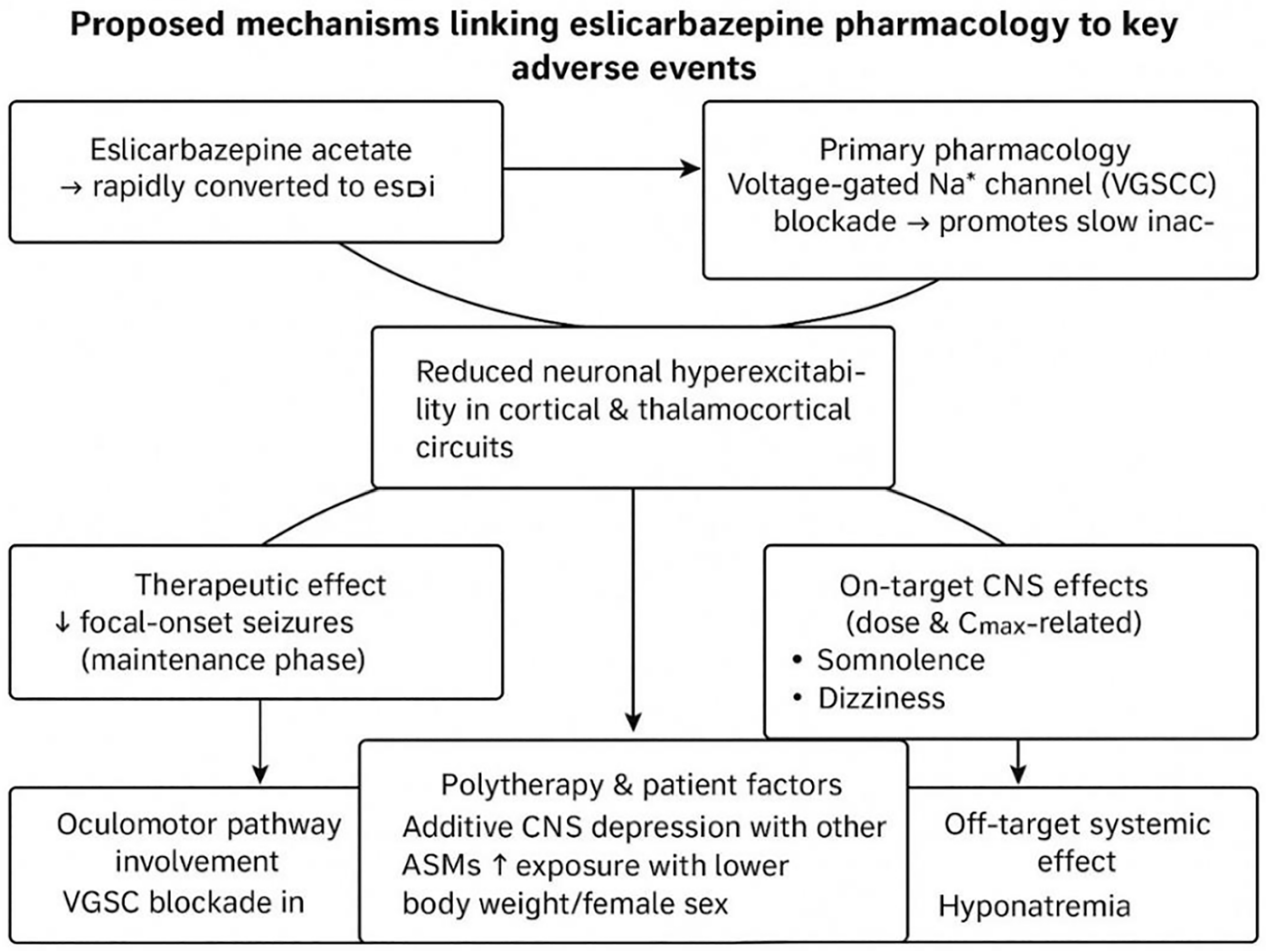
Proposed mechanisms linking eslicarbazepine acetate (ESL) pharmacology to therapeutic effects and adverse events.

Figure 9 shows how a single mechanism of action, consistent with the pharmacological profile of ESL, can result in both therapeutic and adverse effects. By favoring the slow inhibition of VGSCs, ESL diminishes neuronal hyperexcitability by facilitating the gradual inactivation of VGSCs, resulting in a reduction of focal‐onset seizures. But the same mechanism could explain some of the main tolerability problems: diplopia from oculomotor pathway involvement and dose‐dependent somnolence or dizziness as on‐target central nervous system (CNS) effects. In addition, patient‐specific factors (such as polytherapy, body weight, and sex) and off‐target systemic actions, including hyponatremia, may exacerbate these adverse events. This integrative framework emphasizes the importance of finding a balance between efficacy and tolerability when optimizing ESL therapy.

By specifically targeting VGSCs, eslicarbazepine, the active metabolite of ESL, promotes slow inactivation and reduces neuronal hyperexcitability. Higher cumulative exposure (AUC_0–24_) was associated with headaches and dizziness in exposure–safety modeling, whereas higher *C*_max_ (peak plasma levels) was associated with a higher likelihood of somnolence. Interestingly, the risk of somnolence doubled when starting at 800 mg/day as opposed to 400 mg/day. Because ESL is quickly metabolized, periods of maximal CNS effect may coincide with its peak concentration timing, which occurs 1–4 h after the dose. Due to higher drug concentrations per body mass, female sex and body weight are linked to an increased risk of both dizziness and somnolence. Those who had previously taken carbamazepine were more likely to experience dizziness but less likely to experience somnolence, suggesting pharmacologic cross‐tolerance or similar mechanisms (Gidal et al. 2018; Strýček et al. 2024).

SAEs were more common in the treatment group. We defined SAEs as any adverse event that resulted in death, was life‐threatening, required hospitalization, or resulted in persistent or significant disability. The most common SAEs included status epilepticus, convulsions, bronchopneumonia, and device malfunctions such as issues with ventriculoperitoneal shunts. In pediatric populations experiencing focal seizures, SAEs such as pneumonia or complications arising from medical devices (e.g., cerebrospinal fluid shunts) are frequently more credibly linked to the underlying neurological condition, comorbidities, or medical interventions, rather than to the ESL itself. Pooled analysis of pediatric Phase II/III RCTs found no significant difference in SAE incidence between ESL and placebo groups (RR = 1.40; 95% CI: 0.69–2.86; *p* = 0.35). Partial seizures and pneumonia were the most common SAEs (Mintz et al. 2020). ESL medication is unlikely to cause these occurrences. Future safety evaluations should consider systematic categorization of adverse events based on their likely origins, drawing a distinction among those medicine is likely related to and those which are more likely associated with the underlying disease, other medical issues, or a combination of these elements.

Patient comorbidities are more likely to be linked to complications like bronchopneumonia than ESL, according to studies. Neurologic deficiencies, such as hypotonia, dysphagia, and scoliosis, are common in children with refractory focal epilepsy and raise the risk of aspiration and respiratory infections. Since bronchopneumonia is not a known pharmacologic effect of ESL (and happened at comparable or higher rates in non‐ESL groups), the underlying illness, concurrent devices (like shunts), or chance are more likely to be to blame for these cases than the medication itself (Tambucci et al. 2016). In the study by Kirkham, 11.2% patients belonging to the ESL group experienced severe side effects as compared to 8.5% in the placebo group.

ESL was discontinued in 5.2% patients (*n* = 7) compared to 2.3% patients (*n* = 3) in the placebo group due to adverse events. Regarding causality, status epilepticus and convulsions are known effects of antiepileptic drugs (AEDs) and are considered probable adverse effects of ESL. However, events like bronchopneumonia and device malfunctions are unlikely to be caused by the medication, as they are not typically associated with antiepileptic treatment. Therefore, while ESL is effective, the causality of some adverse events remains uncertain and warrants further investigation. Treatment discontinuation due to adverse effects was higher in the ESL group. In the study by Kirkham et al. (Perucca et al. 2020), 5.2% of patients in the treatment group withdrew from the treatment due to an adverse event, compared to 2.3% in the placebo group. Similar trends were observed in other studies, as in the research work by Jóźwiak et al. The author found that the incidence of adverse effects was directly related to the specific drug used by patients, as indicated by the percentage of adverse effects reported. The percentage of serious adverse effects noted when the drug (ESL) is given versus placebo is 3.6% in the ESL group and 5.0% in the placebo group, whereas a publication by Mintz et al. reported an incidence of SAEs in 2.5% of patients from the placebo group. About 5.9% of patients developed SAEs that led to the premature discontinuation of the drug in the study by Mintz et al. Hence, the results indicate that while the treatment is effective, its adverse events might lead to a significant number of patients discontinuing the therapy. Studies included are multicenter, randomized, double‐blind, placebo‐controlled trials. Hence, the findings of this analysis are based on diverse populations, which enhances the generalizability and applicability of the results.

The significant reduction in SSF indicates that ESL as adjuvant therapy for focal seizures (without secondary generalization) is effective as compared to placebo. These results are consistent with findings in adult populations and align with previous evidence on the efficacy of other sodium‐channel modulating ASMs such as oxcarbazepine and carbamazepine. The effective management of seizure disorders can improve quality of life. However, the higher incidence of somnolence, diplopia, and SAEs highlights the need for careful monitoring and management of adverse events. Clinicians should weigh the benefits of seizure reduction against the risk of adverse events and consider individual patient characteristics when prescribing this treatment.

The included studies were multicenter, randomized, placebo‐controlled trials. All the studies were double blinded, which reduces the risk of bias and strengthens the validity of this meta‐analysis. A comprehensive analysis of multiple studies enhances the robustness of the findings.

Inclusion of multiple adverse events in the study gives a detailed safety profile of the treatment. This could help the public health system in devising strategies that should include patient education, regular monitoring, and support systems to manage the adverse effects and optimize treatment outcomes.

Overall, the safety data that have been made public do not warrant an overly rigorous monitoring schedule that exceeds acknowledged standards. Although care is advised (as with any ASM), there is little evidence that ESL has a unique or unanticipated toxicity. For example, clinical trials and post‐market data suggest that hyponatremia with ESL is usually moderate, asymptomatic, and dose‐dependent. Regular electrolyte measurements (e.g., at baseline and regularly during titration) are adequate since only a small number of patients have clinically severe hyponatremia at high dosages. Children's studies have revealed no unusual organ toxicities (Strýček et al. 2024).

According to Zhu et al.’s pooled study, the increased risk of significant adverse events on ESL was caused mostly by predicted occurrences, such as seizure exacerbations, rather than new safety signals. As a result, there is currently insufficient information to warrant a recommendation for “strict” or especially extensive monitoring. ESL treatment should be properly controlled by normal AED monitoring (frequent clinician review, periodic labs), with a focus on symptoms such as excessive tiredness or dizziness (Zhu et al. 2020). To summarize, the harmful effects of ESL on children are typically dose‐related and, with the correct precautions, may be avoided. According to clinical standards, many ASM adverse effects are suggestive of general CNS depression and often improve with dosage modifications. Somnolence and diplopia can be reduced by reducing concomitant sedative medicines or progressively increasing ESL. Similarly, ESL‐associated hyponatremia is generally dose‐dependent and may be avoided in vulnerable patients by not taking exceedingly high doses. Consistent with these data, AE trial terminations were quite uncommon, showing that side effects are usually under control. As a result, ESL's adverse effects are typically manageable and do not outweigh its therapeutic advantages when dosages are adequately titrated and co‐medications are carefully chosen (Strýček et al. 2024).

The short treatment period of ESL in the included RCTs limits the applicability of the findings. Hence, this exploration cannot provide insights into the long‐term efficacy and safety profile of ESL as adjuvant therapy for focal seizures. The majority of the population included in this study belongs to the Caucasian ethnicity, which limits the generalizability of the findings. Two of three included trials were sponsored by a pharmaceutical company that may increase the risk of bias in our findings. A rigorous dose‐response analysis was unfeasible due to the insufficient number of RCTs and the small subgroup sizes within each dose arm (e.g., 20 vs. 30 mg/kg/day).

Subsequent research employing stratified dosing regimens is essential to ascertain the appropriate ESL dosage in the management of pediatric epilepsy and to find the long‐term efficacy and safety profile of ESL as adjuvant therapy for focal seizures. Several important knowledge gaps remain. First, there is a lack of age‐adjusted titration guidelines, and existing trials typically extrapolate from adult dosing (e.g., 10–30 mg/kg) to determine the best dosing strategies for ESL in children. Second, there is a dearth of information on developmental outcomes and long‐term safety. The effects on growth, cognition, or organ function over the years are unknown because the majority of ESL trials have a relatively short follow‐up (weeks to months). Last but not least, research on drug–drug interactions in children is lacking. While adult data indicate that ESL is less auto‐inductive than carbamazepine, more research is required to fully understand the effects of pediatric metabolism and polytherapy. Extension studies and real‐world registries are needed, as reviews highlight that current meta‐analyses are based on short‐term RCTs. Similarly, to close these gaps, research priorities for pediatric epilepsy have specifically called for more comparative and longitudinal studies (Zhu et al. 2020). Larger sample sizes and longer follow‐up periods should be included in future research. Research on alternative dose strategies or adjunct therapies to reduce the adverse effects should be done.

## 5 Conclusion

This study demonstrates that ESL significantly reduces seizure frequency; however, it is also associated with a higher incidence of adverse events such as headache, somnolence, diplopia, nausea, and vomiting. Most of the adverse events were mild to moderate and did not result in treatment discontinuation, SAEs—including severe seizures and device‐related complications—were reported more commonly in the ESL group and led to treatment discontinuation in up to 5.9% of patients. In contrast, 2.3%–2.5% of placebo recipients discontinued treatment for the same reasons. It is important to consider that the occurrence of certain adverse effects, particularly somnolence and diplopia, may be influenced by concomitant use of other AEDs. It is acknowledged that polytherapy is more likely to lead to the development of some specific adverse effects. Evidence suggests that taking ESL with ASMs like lamotrigine or carbamazepine may increase the risk of CNS‐related side effects like somnolence and diplopia, possibly due to pharmacokinetic or pharmacodynamic interactions. This emphasizes the importance of individualized treatment protocols and close supervision when ESL is used as a supplementary therapy.

From our side, we stress the urgency of further investigation, concentrating not only on comparative effectiveness but also on the ESL interaction with other commonly used ASMs. The study will be essential for optimizing the treatment protocols and enhancing the safety and tolerability profiles of antiepileptic therapies for pediatric patients with focal seizures.

## Author Contributions

Every author equally participated in formulating and designing the study. The literature search and screening, as well as the collection and analysis of data, documenting the figures, interpreting the data, and preparing the manuscript, were all carried out by N.B.D. and A.A. All authors read and commented on the manuscript. All authors had full access to all the data in the study and had final responsibility for the decision to submit for publication.

## Funding

The authors have nothing to report.

## Ethics Statement

The authors have nothing to report.

## Consent

The authors have nothing to report.

## Conflicts of Interest

The authors declare no conflicts of interest.

## Supporting information




**Supplementary Material**: brb371036‐sup‐0001‐SuppMatt.docx

## Data Availability

The dataset supporting the conclusion of this article is included in this article. We did not generate any new data for this study. All data used are available from the publications or authors of included studies.
