# Supplementary material for: Safety and Efficacy of Eslicarbazepine Acetate in Children Diagnosed With a Focal Seizure Disorder: A Systematic Review and Meta‐Analysis
Source: Brain Behav. 2025 Nov 24;15(12):e71036. doi: 10.1002/brb3.71036 (PMC12643949; doi:10.1002/brb3.71036)
Supplement: Supplementary file 1 — Supplementary Material: brb371036‐sup‐0001‐SuppMatt.docx [file BRB3-15-e71036-s001.docx]

**QUALITY ASSESSMENT:**


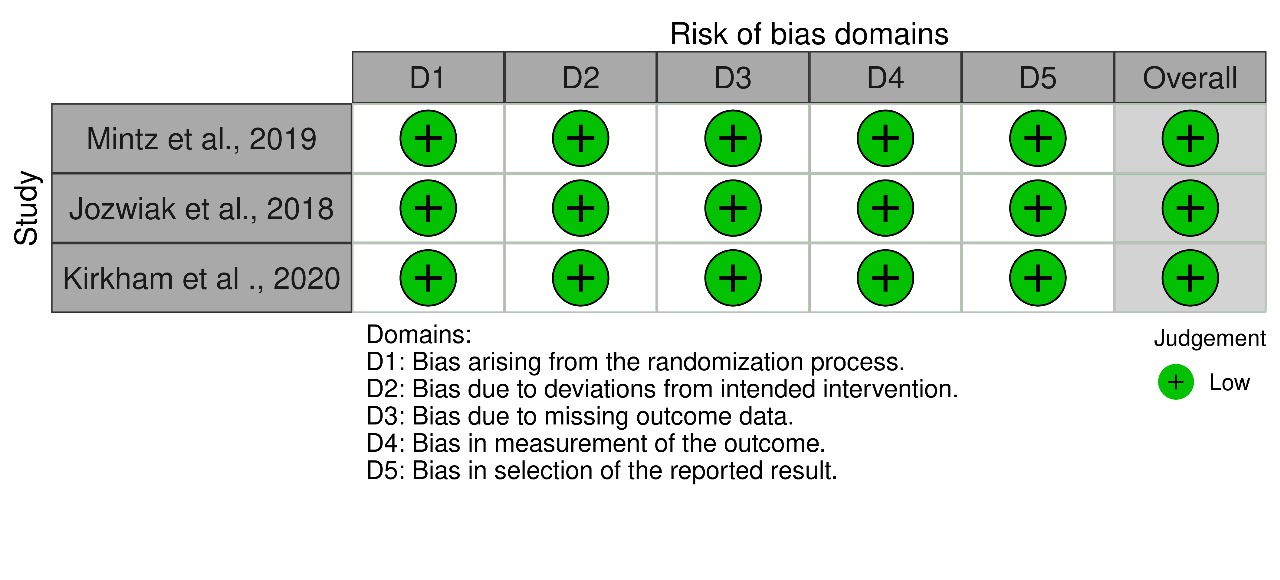


*- Traffic Light Plot* (Figure 1)


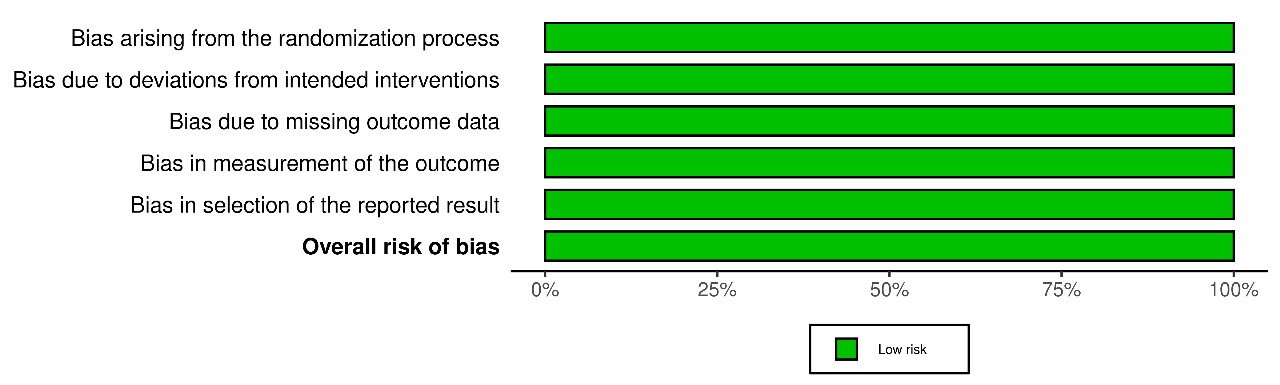


*- Weighted Bar Plot* (Figure 2)

**PRISMA FLOW DIAGRAM (Figure 3)**

**Previous studies**

**Identification of new studies via databases and registers**

Studies included in previous version of review (n = 2)

Reports of studies included in previous version of review (n =2)

Records identified from*:

Databases (n =937)

[Pubmed =5]

[Cochrane =11]

[Google scholar =921]

Registers (n =0)

Records removed *before screening*:

Duplicate records removed (n =4 )

Records marked as ineligible by automation tools (n =0)

Records removed for other reasons (n =0)

**Identification**

Reports sought for retrieval

(n =5)

Records screened

(n =933)

Records excluded**

(n =928)

Reports not retrieved

(n =0)

**Screening**

Reports excluded:

(n = 4)

(studies not aligned to pico)

Reports assessed for eligibility

(n =1)

New studies included in review

(n =1)

Reports of new included studies

(n =1)

**Included**

Total studies included in review

(n =3)

Reports of total included studies

(n =3)

*Consider, if feasible to do so, reporting the number of records identified from each database or register searched (rather than the total number across all databases/registers).

**If automation tools were used, indicate how many records were excluded by a human and how many were excluded by automation tools.

**Search strategy:**

P: Children (aged 0-18 years) diagnosed with epilepsy or seizure disorders

I: Eslicarbazepine acetate

C: Placebo

O: Long-term therapeutic effects (e.g., seizure control, reduction in seizure frequency, quality of life improvements)

**(Table 1)**

| **DATABASES** | **SEARCH STRATEGY** | **Filters applied** | **RESULTS** |
| --- | --- | --- | --- |
| PubMed | ("Child"[MeSH Terms] OR "child, preschool"[MeSH Terms] OR "Pediatrics"[MeSH Terms] OR "Infant"[MeSH Terms] OR "child, hospitalized"[MeSH Terms]) AND ("eslicarbazepine acetate"[Title/Abstract] OR "ESL"[Title/Abstract] OR "eslicarbazepine acetate"[Title/Abstract]) AND ("epilepsies, partial"[MeSH Terms] OR "Seizures"[MeSH Terms] OR "epilepsy, partial, sensory"[MeSH Terms]) | Randomized control trial, articles in English language and till year 2024 | 05 |
| Cochrane | **#1**: MeSH descriptor: [Child] explode all trees  **#2:** eslicarbazepine acetate  **#3:** MeSH descriptor: [Epilepsy] explode all trees  **#4:** MeSH descriptor: [Seizures] explode all trees  **#5:** MeSH descriptor: [Placebos] explode all trees  **#6:** MeSH descriptor: [Treatment Outcome] explode all trees  **#7:** #1 AND #2 AND #3 OR #4 AND #5 AND #6 | Randomized control trial, articles in English language and till year 2024 | 82499  265  3515  1782  27215  203814  11 |
| Google Scholar | "children" OR "pediatrics" AND "epilepsy" OR "seizures" AND "eslicarbazepine  acetate" AND "placebo" AND "efficacy" OR "treatment outcome" | Randomized control trial, articles in English language and till year 2024 | 921 |

Table 2: Patient Demographics and Adverse Event Profile by Treatment Arm

| **Study participants’ characteristics** | **Sergiusz Jóźwiak,2018** | | **Fenella Kirkham,2020** | | **Mark Mintz, MD1,2019** | |
| --- | --- | --- | --- | --- | --- | --- |
| Treatment arms | **Placebo** | **ESL** | **Placebo** | **ESL** | **Placebo** | **ESL** |
| Patients, n | 39 | 78 | 129 | 134 | 160 | 202 |
| Male sex, % | 65.0 | 56.6 | 48.1 | 47.8 | (52.5) | 49.5 |
| Age, years, mean | 11.7 | 11.7 | 9.5 | 9.9 | 12.5 | 12.5 |
| Caucasian, % | 97.5 | 100 | 90 | 90 | 97 | 97 |
| Concomitant AEDs, % | | | | | | |
| percentage of adverse events | 41 | 47.5 | 72.9 | 83.6 | 65.6 | 67.8 |
| percentage of patients with vomiting | 2.5 | 6 | 4.7 | 7.7 | 5 | 7.9 |
| percentage of patients with somnolence | 5 | 6 | 24.8 | 41.8 | 5 | 9.4 |
| percentage of patients with respiratory tract infection | 5.4 | 9.4 | 5.4 | 5.2 | 3.9 | 4.5 |
| percentage of patients with pyrexia | 5 | 1.2 | 5.4 | 7.5 | 2.5 | 2 |
| percentage of patients with nausea | 5 | 2.4 | 0.8 | 5.2 | 1.9 | 5 |
| percentage of patients with dizziness | 2.5 | 3.6 | 1.6 | 3.7 | 2.5 | 4.5 |
| percentage of patients with diplopia | 0 | 3.6 | 1.6 | 6 | 1.3 | 6.4 |
| percentage of patients with headache | 15 | 9.6 | 6.2 | 13.4 | 11.3 | 13.9 |
| percentage of patients with TEAE | 47.5 | 41 | 72.9 | 83.6 | 65.6 | 67.8 |

Table 3: Study characteristics

| **Studies** | **Study design** | **Main inclusion criterion** | **Treatment arms** |
| --- | --- | --- | --- |
| Mark Mintz, MD1,2019 | pooled analysis of data from two randomized, double-blind, placebo-controlled clinical trials of adjunctive eslicarbazepine acetate (ESL) in pediatric patients aged 4-17 years with focal seizures. | Patients previously treated with 3 AEDs without seizure control, and currently taking 1 or 2 AEDs | two treatment arms in this study were placebo and eslicarbazepine acetate (ESL) as adjunctive therapy. |
| Fenella Kirkham,2020 | a multicenter, phase III, randomized, double-blind, placebo-controlled, parallel-group study to evaluate the efficacy of eslicarbazepine acetate (ESL) as adjunctive therapy in children and adolescents with refractory focal-onset seizures. The study consisted of an 8-week baseline period, followed by a 6-week double-blind titration period, a 12-week double-blind maintenance period, a 4-week taper-off period, and a 4-week follow-up period. There was also a 48-week open-label extension period. | Age between 2-18 years old Diagnosed with epilepsy for at least 6 months prior to enrollment  Having focal-onset seizures (FOS) with at least 4 seizures in the month before enrollment Receiving 1-2 antiepileptic drugs (AEDs), except oxcarbazepine Randomized to either ESL or placebo, stratified by age group (2-6 years, 7-11 years, 12-18 years) | The treatment arms in this study were eslicarbazepine acetate (ESL) and placebo. |
| Sergiusz Jóźwiak,2018 | a multicenter, phase II, randomized, double-blind, placebo-controlled, parallel study. It had two parts: Part I was a 12-week double-blind period with a 4-week up-titration and 8-week maintenance period, followed by Part II, a one-year open-label extension period. | Age between 6-16 years old  Diagnosed with epilepsy for at least 12 months prior to enrollment  Had at least 2 focal-onset seizures (≥4 in the month before enrollment) Receiving 1-2 antiepileptic drugs (except oxcarbazepine) | The treatment arms in this study were ESL (eslicarbazepine acetate) and placebo. Patients were randomized 2:1 to receive either ESL or placebo |

Table 4: Baseline characteristics

| Author name | Intervention | No. of patients | | Age (years), (SD) | | Male | | Female | | Height, cm; median (range) | | Body weight, kg; median (range) | | Number of baseline AEDs used n (%)  1 | | Number of baseline AEDs used n (%)  2 | | Number of baseline AEDs used n (%)  3 | |
| --- | --- | --- | --- | --- | --- | --- | --- | --- | --- | --- | --- | --- | --- | --- | --- | --- | --- | --- | --- |
|  |  | Placebo | Intervention | Placebo | Intervention | Placebo | Intervention | Placebo | Intervention | Placebo | Intervention | Placebo | Intervention | Placebo | Intervention | Placebo | Intervention | Placebo | Intervention |
| MD et al.,2019 | Eslicarbazepine Acetate | 160 | 202 | 10 (4-17) | 11 (4-17) | 84 | 100 | 76 | 102 | 143 (95-190) | 147 (89-192) | 37.0 (12.0-121.0) | 40.5 (12.0-99.0) | 37 (23.1) | 61 (30.2) | 112 (70.0) | 128 (63.4) | 11 (6.9) | 13 (6.4) |
| Jóźwiak et al.,2018 | eslicarbazepine acetate | 40 | 83 | 11.6 (2.8) | 11.8 (3.1) | 26 | 47 | 14 | 36 |  |  |  |  | 19 (47.5) | 44 (53.0) | 18 (45.0) | 37 (44.6) |  |  |
| Kirkham et al.,2020 | eslicarbazepine acetate | 129 | 134 | 9.5 (3.9) | 9.9 (4.2) | 62 | 64 | 67 | 70 |  |  |  |  | 25 (19.4) | 21 (15.7) | 94 (72.9) | 98 (73.1) | 10 (7.8) | 15 (11.2) |

Table 5: Outcomes

| Author name | Adverse effects | | | | | | | | | | | | | |
| --- | --- | --- | --- | --- | --- | --- | --- | --- | --- | --- | --- | --- | --- | --- |
|  | Patients with any TEAE | | Headache | | Somnolence | | Vomiting | | Nausea | | Diplopia | | Dizziness | |
|  | Placebo | Intervention | Placebo | Intervention | Placebo | Intervention | Placebo | Intervention | Placebo | Intervention | Placebo | Intervention | Placebo | Intervention |
| MD et al.,2019 | 105/160 (65.6) | 137/202 (67.8) | 18/160 (11.3) | 28/202 (13.9) | 8/160 (5.0) | 19/202 (9.4) | 8/160 (5.0) | 16/202 (7.9) | 3/160 (1.9) | 10/202 (5.0) | 2/160 (1.3) | 13/202 (6.4) | 4/160 (2.5) | 9/202 (4.5) |
| Jóźwiak et al.,2018 | 19/40 (47.5) | 34/83 (41.0) | 6/40 (15.0) | 8/83 (9.6) | 2/40 (5.0) | 5/83 (6) | 1/40 (2.5) | 5/83 (6) | 2/40 (5.0) | 2/83 (2.4) | 0/40 | 3/83 (3.6) | 1/40 (2.5) | 3/83 (3.6) |
| Kirkham et al.,2020 | 94/129 (72.9) | 112/134 (83.6) | 8/129 (6.2) | 18/134 (13.4) | 6/129 (4.7) | 15/134 (11.2) | 8/129 (6.2) | 8/134 (6.0) | 1/129 (0.8) | 7/134 (5.2) | 2/129 (1.6) | 8/134 (6.0) | 2/129 (1.6) | 5/134 (3.7) |

Table 6: Adverse effects for placebo and intervention

| **Adverse event** | **No. of participants (ESL/placebo)** | **Risk ratio M–H, fixed (95% CI)** | **p value** |
| --- | --- | --- | --- |
| Headache | 419/339 | 1.26[0.70,2.26] | 0.44 |
| Somnolence | 419/339 | 1.95[1.11,3.43] | 0.02 |
| Vomiting | 419/339 | 1.35[0.74,2.44] | 0.33 |
| Nausea | 419/339 | 2.06[0.54,7.90] | 0.29 |
| Diplopia | 419/339 | 4.34[1.60,11.78] | 0.004 |
| Dizziness | 419/339 | 1.88[0.79,4.49] | 0.15 |
